# Trichuris trichiura in a post-Colonial Brazilian mummy

**DOI:** 10.1590/0074-02760140367

**Published:** 2015-02

**Authors:** Rafaella Bianucci, Eduardo J Lopes Torres, Juliana MF Dutra Santiago, Luis F Ferreira, Andreas G Nerlich, Sheila Maria Mendonça de Souza, Valentina Giuffra, Pedro Paulo Chieffi, Otilio Machado Bastos, Renata Travassos, Wanderley de Souza, Adauto Araújo

**Affiliations:** 1 Laboratory of Physical Anthropology, Department of Public Health and Paediatric Sciences, University of Turin, Turin, Italy; 2 Department Biosciences, Centre for Ecological and Evolutionary Synthesis, University of Oslo, Oslo, Norway; 3 Laboratoire d’Anthropologie Bio-Culturelle, Droit, Etique & Santé, Unités Mixtes de Recherche 7258, Faculté de Médecine de Marseille, Marseille, France; 4 Laboratório de Helmintologia Romero Lascasas Porto, Departamento de Microbiologia, Imunologia e Parasitologia, Faculdade de Ciências Médicas, Universidade do Estado do Rio de Janeiro, Rio de Janeiro, RJ, Brasil; 5 Laboratório de Ultraestrutura Celular Hertha Meyer, Instituto de Biofísica Carlos Chagas Filho/Instituto Nacional de Ciência e Tecnologia em Biologia Estrutural e Bioimagem, Universidade Federal do Rio de Janeiro, Rio de Janeiro, RJ, Brasil; 6 Laboratório de Paleoparasitologia, Escola Nacional de Saúde Pública Sérgio Arouca-Fiocruz, Rio de Janeiro, RJ, Brasil; 7 Institute of Pathology, Klinikum München-Bogenhausen, Munich, Germany; 8 Division of Paleopathology, Department of Translational Research on New Technologies in Medicine and Surgery, University of Pisa, Pisa, Italy; 9 Departamento de Patologia, Faculdade de Ciências Médicas da Santa Casa de São Paulo, São Paulo, SP, Brasil; 10 Departamento de Microbiologia e Parasitologia, Centro de Ciências da Saúde , Universidade Federal Fluminense, Niterói, RJ, Brasil; 11 Divisão de Biologia Estrutural, Diretoria de Metrologia Aplicada às Ciências da Vida, Instituto Nacional de Metrologia, Qualidade e Tecnologia, Duque de Caxias, RJ, Brasil

**Keywords:** whipworm, soil-transmitted helminthiasis, *Maculo* syndrome

## Abstract

Trichuris trichiura is a soil-transmitted helminth which is prevalent in warm, moist, tropical and subtropical regions of the world with poor sanitation. Heavy whipworm can result either in Trichuris dysenteric syndrome - especially in children - or in a chronic colitis. In heavy infections, worms can spread proximally and may cause ileitis. Here we provide first microscopic evidence for a T. trichiura adult worm embedded in the rectum of a post-Colonial Brazilian adult mummy. During Colonial and post-Colonial times, many European chroniclers described a parasitic disease named Maculo whose symptomatology coincides with heavy helminthiasis. Based on our findings and on comparison of ancient textual evidence with modern description of heavy whipworm, we feel confident in considering that the two syndromes are expressions of the same pathological condition.

*Trichuris trichiura* is a soil-transmitted helminth which is prevalent in warm, moist, tropical and subtropical regions of the world with poor sanitation (e.g., Sub-Saharan Africa, India, China, a large part of Asia, Latin America and Caribbean and Middle Eastern Crescent) (Azira & Zeehaida 2012). The infection is acquired by ingestion of contaminated water or foods and is mostly asymptomatic. However, when it progresses from light to heavy infection, specific diseases manifest.

Heavy whipworm infection may cause the insurgence of the so-called *Trichuris* dysenteric syndrome (TDS) especially in young children; TDS is characterised by mucoid diarrhoea, rectal bleeding and rectal prolapse complicated with severe bacterial secondary infection (Cooper et al. 1992). In adults, heavy trichuriasis can result either in TDS or in a chronic colitis that shares many clinical features with other bowel diseases such as Crohn disease or ulcerative colitis; in heavy infections, worms can also spread proximally and may cause ileitis (Long et al. 2012).

Here we report on a case of *T. trichiura* adult worm infection in a late XVIII-early XIX century naturally mummified body (mummy A74, adult male) unearthed from the topsoil of Itacambira's church [state of Minas Gerais (MG), Brazil].

A previous study carried out on a coprolite from mummy A74 had already revealed the presence of few *T. trichiura* eggs in its faeces although the exact burden of the infection could not be established due to the eggs' poor state of preservation (Confalonieri et al. 1981). Recently, paleoparasitological investigations were further expanded.

Biopsies were taken from rectum of the mummy A74 and subjected to histological investigations. A longitudinal fragment of 8 cm of the rectum wall was sampled. After rehydration in Sandison solution for five days, samples were fixed for 24 h in 10% buffered formalin, dehydrated and embedded in paraffin blocks. The cuts were made in 3 µm thick sections. The paraffin sections were histochemically counterstained with haematoxylin and eosin stain.

Light microscopy showed in the tissue of the rectal wall the presence of five peculiar round structures, which ranged from 42.6 μm in length to 56.4 μm in breadth, embedded in mummy A74's rectal tissue (B in Figure). Through scanning electron microscopy, these structures were identified as transversal cuts of the anterior region of a *T. trichiura* adult worm (E, F in Figure). The spacing between these structures was 48 µm, thus suggesting that these were different sections of a single worm. The diagnosis was achieved by comparing the cuticular structures seen in the mummy biopsy with those observed in mice experimentally infected with *Trichuris muris* (A, C, D in Figure).

**Figure:**
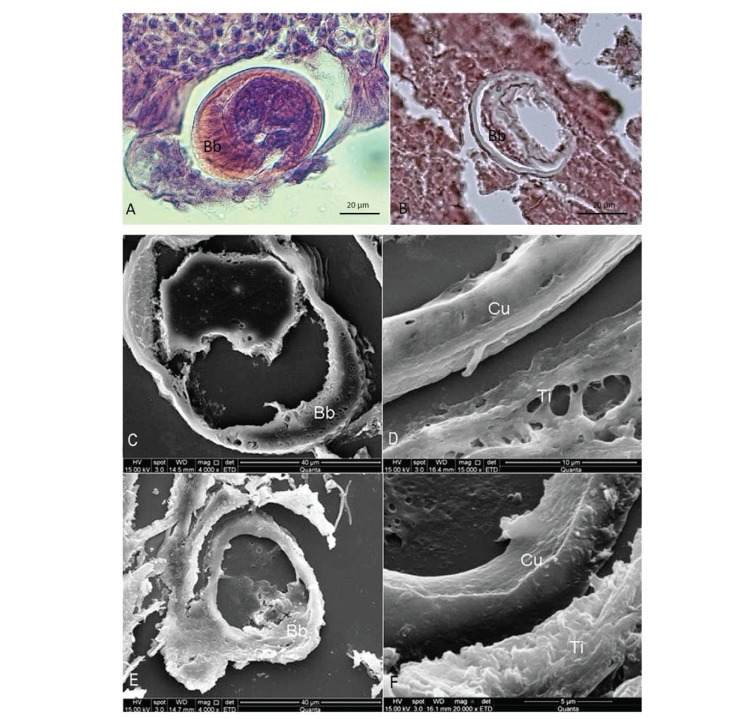
Upper row: light microscopy of histological section. A: anterior region of *Trichuris* *muris *inserted in the large intestine of modern laboratory mouse, showing the bacillary band region (Bb) on the nematode cuticle (Cu) as comparison for archaeological material; B: anterior region of *Trichuris trichiura *inserted in the terminal portion of the rectum of human mummified tissue under study, showing the typical cuticular surface with the Bb. Lower row: scanning electron microscopy of histological section; C: anterior region of *T.* *muris *inserted in the large intestine of modern laboratory mouse, showing the Bb; D: detail of the Cu and host tissue, forming a epithelial tunnel (Ti) Bb on the nematode Cu; E: anterior region of *T. trichiura *inserted in the terminal portion of large human mummified intestinal tissue, showing the Cu with adjacent secondary Bb; F: *T. trichiura* Cu and host tissue (Ti).

Adult *T. trichiura* worms introduce their anterior region in the intestinal mucosa, mainly in the caecum where they prevalently reside. However, during severe *T. trichiura* infections, worms colonise the entire gross intestine down to the rectal region where they cause tissue damage, oedema and secondary bacterial invasion (Gilman et al. 1976, Stephenson et al. 2000).

The presence of a single *T. trichiura *adult worm embedded in the rectum of individual A74 is consistent with a heavy whipworm infection. It is reasonable to hypothesize that a higher number of adult worms might have been originally present in individual A74's rectum at the time of his death; however, poor overall preservation conditions of the corpse prevented us from identification of further worms.

Previous recovery of coprolites in mummy A74 (Confalonieri et al. 1981) is inconsistent with a TDS diagnosis; it is consistent, instead, with chronic colitis (Long et al. 2012) whose long-term effects on the health conditions of this man remain unknown.

As a matter of fact, mummy A74 dates back to the time of gold and diamond expeditions in the most remote areas of Brazil (e.g., MG). Both nutritional and sanitary conditions among the people who partook these expeditions were extremely poor and people were, therefore, exposed to several infections caused by different parasites.

Whereas *T. trichiura* eggs have been abundantly found in coprolites of ancient individuals from all continents dating both to prehistoric and historic periods (Gonçalves et al. 2003, Reinhard et al. 2008, Jiménez et al. 2012, Morrow et al. 2014, Rácz et al. 2015), the presence of *T. trichiura* adult worms in mummies has never been reported in paleoparasitological literature.

During Colonial and post-Colonial periods, many European chroniclers described a parasitic disease named *Maculo* or *Mal del Culo *(disease of the anus) or *doença do bicho* (disease of the bug); this disease was characterised by "rectal inflammation, fetid mucous elimination, ulcerations and bloody diarrhoea" accompanied, sometimes, by rectal prolapse (Sigaud 1844).

First descriptions of the disease and of a tentative treatment (a mixture of pepper powder, crushed with tobacco, gunpowder and other herbs, introduced through the anus by an enema or applied externally) were given between the end of the XVI century [de Sousa (1851) , written in 1587] and the first half of the XVII century AD (de Abreu 1623, Piso 1648).

The *Maculo* syndrome, which was enhanced by poor nutrition and unsanitary conditions, is claimed to have caused hundreds of deaths in Colonial and post-Colonial Brazil (Rezende 2003). Based on our findings and on comparison of ancient textual evidence with modern description of TDS, we feel confident in considering the two syndromes as the expressions of the same pathological condition.
