# Erratum: Trichuris trichiura in a post-Colonial Brazilian mummy

**DOI:** 10.1590/0074-02760140367ER

**Published:** 2015-03-24

Vol. 110 (1): 145-147, 2015.

p. 145

Rafaella Bianucci^1,2,3/+^, Eduardo J Lopes Torres^4,5^, Juliana MF Dutra Santiago^6^, Luis F Ferreira^6^, Andreas G Nerlich^7^, Sheila Maria Mendonça de Souza^6^, Valentina Giuffra^8^, Pedro Paulo Chieffi^9^, Otilio Maria Bastos^10^, Renata Travassos^5,11^, Wanderley de Souza^5,11^, Adauto Araújo^6^

**should read:**

Raffaella Bianucci^1,2,3/+^, Eduardo J Lopes Torres^4,5^, Juliana MF Dutra Santiago^6^, Luiz Fernando Ferreira^6^, Andreas G Nerlich^7^, Sheila Maria Mendonça de Souza^6^, Valentina Giuffra^8^, Pedro Paulo Chieffi^9^, Otilio Machado Bastos^10^, Renata Travassos^5,11^, Wanderley de Souza^5,11^, Adauto Araújo^6^

